# Breed-Specific Clinical Features, Diagnostic Findings, and Outcome of Presumptive Meningoencephalomyelitis of Unknown Origin in 27 French Bulldogs †

**DOI:** 10.3390/vetsci12020083

**Published:** 2025-01-23

**Authors:** Evelina Burbaite, Erica Fiorentino, Greta Galli, Antonella Gallucci, Federica Tirrito, Gualtiero Gandini, Samuel Okonji, Marika Menchetti

**Affiliations:** 1 Neurology and Neurosurgery Division, San Marco Veterinary Clinic, 35030 Veggiano, Italy; evelina.burbaite.vet@gmail.com (E.B.); erica.fiorentino@sanmarcovet.it (E.F.); greta.galli@sanmarcovet.it (G.G.); 2 Dr. L. Kriaučeliūnas Small Animal Clinic, Faculty of Veterinary, Veterinary Academy, Lithuanian University of Health Sciences, 47181 Kaunas, Lithuania; 3 Neurological Veterinary Center La Fenice, 09047 Selargius, Italy; antonella.gallucci@tiscali.it; 4 AniCura Istituto Veterinario di Novara, 28060 Novara, Italy; federica.tirrito@yahoo.it; 5 Clinica Neurologica Veterinaria NVA, 20148 Milano, Italy; 6 Department of Veterinary Medical Science, University of Bologna, 47181 Bologna, Italy; gualtiero.gandini@unibo.it (G.G.); samuel.okonji@unibo.it (S.O.)

**Keywords:** meningoencephalomyelitis, magnetic resonance imaging, cerebrospinal fluid, dog, veterinary neurology

## Abstract

Meningoencephalomyelitis of unknown origin (MUO) is an inflammatory central nervous system disease affecting the meninges, brain, and spinal cord. Interest in this condition has been growing rapidly over the last decade, but information on the pathophysiology and evolution of the disease is still lacking. This study aimed to assess and evaluate the clinical findings, magnetic resonance imaging (MRI) features, and survival time in affected French Bulldogs (FBs). A total of 27 client-owned FBs met the inclusion criteria. The highest percentage of animals were affected by acute blindness and cervical pain, representing 25.9% (*n* = 7) each. Via MRI, 44.4% of FBs showed signs of perilesional parenchymal brain edema. Midline shift was observed in 11.1% of dogs, and 7.4% displayed foramen magnum cerebellar herniation. The cerebrospinal fluid examination was considered normal in 34.8% of dogs. Five (18.5%) dogs died during the treatment. Close to two-thirds (63.0%) were still alive during the study data collection (median survival time, 775 days), displaying an overall good survival time. The presence of epileptic seizures, midline shift, and relapse of clinical signs were factors associated with higher mortality rate (*p* < 0.05) and might be prognostic factors for worse survival in French Bulldogs with MUO.

## Simple Summary

Meningoencephalitis/meningoencephalomyelitis of unknown origin (MUO) is a term used to describe the most likely immune-mediated, inflammatory diseases of the brain and/or spinal cord. The number of patients diagnosed with MUO has been growing over the years; therefore, this condition is becoming a pathology of increasing importance among veterinary neurologists. However, data are lacking for understanding the different presentations and evolution of the disease. It is unclear whether the histologically confirmed subtypes of the disease are separate entities or stages of the same pathology, which could be the reason why several affected dog breeds present differently. The aim of this study was to analyze MUO in the French Bulldog breed and present its characteristics, diagnostic findings, and outcomes. It was found that French Bulldogs present with MUO in a similar manner to other affected breeds, and that certain diagnostic imaging characteristics could be potential prognostic factors for a better or worse outcome of the disease.

## 1. Introduction

Meningoencephalomyelitis of unknown origin (MUO) is an umbrella term, used to describe inflammatory, most likely immune-mediated diseases of the central nervous system (CNS). Histologically recognized subtypes of MUO include granulomatous meningoencephalitis (GME), necrotizing meningoencephalitis (NME), and necrotizing leukoencephalitis (NLE), all presenting with different histological, and immunohistochemical features [1,2]. The exact pathophysiology of MUO remains unclear [3]. Apart from a possible genetic predisposition, it is assumed that a certain stimulus factor might play a role [4]. Antigen agents such as infectious and environmental triggers can activate CNS autoreactive cells and lead to inflammatory processes. During the disease, excreted proinflammatory cytokines cause multiple injuries, leading to neurotoxicity, astrogliosis, and apoptosis [4].

The diagnosis of MUO is reached by performing an extensive diagnostic protocol and applying the principle of exclusion. MUO is assumed in cases where advanced imaging of CNS and cerebrospinal fluid (CSF) examination are suggestive of inflammation in the absence of any infectious agent [4,5]. Definitive diagnosis is only reached after histopathological examination of the affected brain, meningeal, or spinal cord tissue; therefore, the exact subtype is only ascertained after performing the biopsy or post-mortem examination [4,6].

Recent research in veterinary medicine has brought up doubts regarding whether the three known subtypes are different entities or just stages of the same disease [7]. Nevertheless, it seems that MUO might exhibit breed-specific patterns [1]. After performing post-mortem histopathological studies, it was noted that Pugs are most likely to be affected by NME, and Yorkshire Terriers are most susceptible to NLE [1,2,8,9]. On the other hand, GME affects mostly small female dogs [10]. Considering this, it may be useful to investigate breed-specific findings of MUO instead of analyzing all the dogs that are affected by MUO.

The French Bulldog (FB) is a breed with a tendency to exhibit a considerable number of neurological pathologies. It is reported that around 18.7% of all admitted FBs show neurological signs [11]. In a recent study, MUO represented 25.0% of all the encephalopathies in this breed [11]. Based on the current literature, the FB is most likely to be affected by necrotizing leukoencephalitis, and its pattern is similar to breed-specific necrotizing encephalitis of Yorkshire Terriers [8,12].

The aim of this study was to analyze the specific characteristics of MUO in a FB dog population and to compare the findings with those in the current veterinary medicine literature.

## 2. Materials and Methods

### 2.1. Case Selection and Data Collection

The data were collected retrospectively from four referral veterinary clinics.

Inclusion criteria for the diagnosis of presumed MUO in FBs were adapted according to the study of Granger et al. 2010 [10], i.e., (a) dogs older than 6 months; (b) neurological signs compatible with CNS involvement; (c) the presence of T2-weighted (T2W) hyperintense, single/multiple, diffuse, and intra-axial lesions on magnetic resonance imaging (MRI); (d) when available, a CSF exam was considered indicative of MUO if, along with the clinical and MRI presentation, it showed a total nucleated cell count (TNCC) > 5 cell/μL; (e) when available, negative infectious disease testing results.

CSF was considered abnormal if it exceeded the reference range of TNCC up to 5 cells/µL, as previously proposed [10].

For inclusion, clinical records were required to describe the medication administered (name, dosage, and duration of therapy). The information on follow-up and the outcome of the disease were collected, when available. Gathered information included animal age, weight, sex, signalment, clinical and neurological examination findings, diagnostic imaging (MRI) and laboratory (CSF, serologic tests) findings, treatment protocol and duration, and information regarding relapses. In living patients, follow-up examination (both during consultations and via telephonic interviews), data were collected.

Exclusion criteria included lack of medical records, lack of MRI study, and evidence of concomitant diseases (i.e., neoplasia) or other inflammatory CNS diseases (eosinophilic CSF). Lastly, dogs with records of previous anti-inflammatory treatment for a suspected MUO were excluded from the study.

### 2.2. Diagnostic Protocol

Neurological examinations were performed or supervised by a board-certified neurologist. For all included dogs, the MRI was performed using low-field or high-field MRI (0.3 Tesla (T) Hitachi Airis II [Fujifilm, Tokyo, Japan], 3 T Siemens Skyra [Siemens Healthineers, Erlangen, Germany], 0.4 T Aperto Lucent [Fujifilm, Tokyo, Japan], 0.22 T Mr.J [Paramed, Markham, ON, Canada], and 1.5 T Vantage Elan [Canon Medical Systems, Tochigi, Japan]). MRI was performed on dogs under general anesthesia and included image acquisitions of the head and cranial portions of the neck. The spinal cord was examined if meningomyelitis was suspected. The required MRI sequences to consider the study complete included sagittal and transverse T2-weighted (T2-W), transverse fluid-attenuated inversion recovery (FLAIR) (for studies of the brain), short tau inversion recovery (STIR) (for studies of the spinal cord), transverse T1-weighted (T1-W), and T1-W post-contrast medium injection images. Dogs were considered to have a severe form of MUO if severe brain edema was observed. Severe edema is diagnosed as the results of an MRI study which are indicative of severe mass effect, obliteration of prosencephalic sulci, midline shift, or brain herniation. The mass effect was described as a displacement of regional brain structures, and midline shift was described as displacement of the midline.

### 2.3. Statistical Analysis

Data collection and statistical analysis were performed using Microsoft^®^ Excel 2019 and IBM SPSS Statistics^®^ Edition 29 software [IBM, Armonk, NY, USA]. The data normality was evaluated using the Shapiro–Wilk test. Normal deviates, which are quantitative indicators, are presented as mean ± standard deviation (SD). Variables which are not distributed normally are described using the median and interquartile range (IQR). Relationships between FB groups were evaluated using the Chi-square (χ^2^) test. A confidence interval (CI) of 95% was used in the statistical analysis. The data were considered statistically significant if the *p*-value < 0.05.

## 3. Results

### 3.1. Study Population

A total of 27 dogs that met the inclusion criteria were included in the study; 51.9% were males (*n* = 14), and 48.1% were females (*n* = 13). The mean age at the onset of clinical signs in dogs was 3.79 (±2.4) years. The mean weight was 12.4 (±2.6) kg. When comparing the variables, all the data were evenly distributed between sexes.

Seizures, lesion type (focal/multifocal), different lesion localizations (forebrain/spinal cord/brainstem/optic tracts), signal intensity changes on MRI, and contrast uptake were equally common between sexes; the relationships were not statistically significant (*p* > 0.05).

### 3.2. Clinical Signs at Presentation

The clinical signs at the presentation are summarized in Table 1. The neurologic signs at presentation varied greatly. The highest percentage of animals were affected by acute blindness and cervical pain, each representing 25.9% (*n* = 7). Epileptic seizures were a presenting sign in 18.5% (*n* = 5) of dogs, yet 25.9% (*n* = 7) of dogs in total developed seizures as an additional clinical sign during the course of the disease. Dogs that showed epileptic seizures as a presenting clinical sign were more likely to have a multifocal MUO lesion (*n* = 4/5, 80.0%), and the majority of dogs that displayed a focal MUO lesion did not experience seizures at presentation (*n* = 8/9, 88.9%); this result was not statistically significant (*p* > 0.05).

### 3.3. Magnetic Resonance Imaging Characteristics and CSF Analysis

The lesion distribution within the CNS parenchyma is summarized in Table 2. In two-thirds of dogs (*n* = 18), the lesions were described as multifocal, and in nine dogs (33.3%), they were focal. Two-thirds (*n* = 18) of FBs displayed detectable MUO lesions in the forebrain. In 40.7% of dogs (*n* = 11), the spinal cord, and in 37.0% of dogs (*n* = 10), the brainstem was affected. More than half (51.9%, *n* = 14) of the French Bulldogs exhibited MRI lesions affecting both the white and gray matter of the brain. In 25.9% (*n* = 7), the lesions selectively involved the gray matter, and in 11.1% (*n* = 3), they affected the white matter.

**Figure 1:**
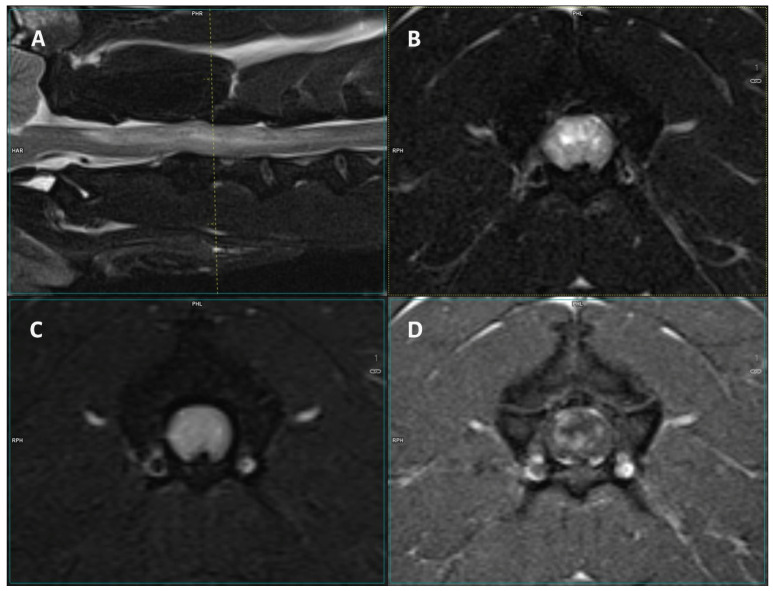
Images of a 3-T MRI study of a 5-year-old male French Bulldog. (**A**) Mid-sagittal T2-W, (**B**) T2-W transverse, (**C**) FLAIR, and (**D**) T1 post-contrast transverse images of the cervical spinal cord. At the level of C2–C4, there is a diffuse, cloudy T2-W and FLAIR hyperintensity that displays inhomogeneous contrast enhancement.

**Figure 2:**
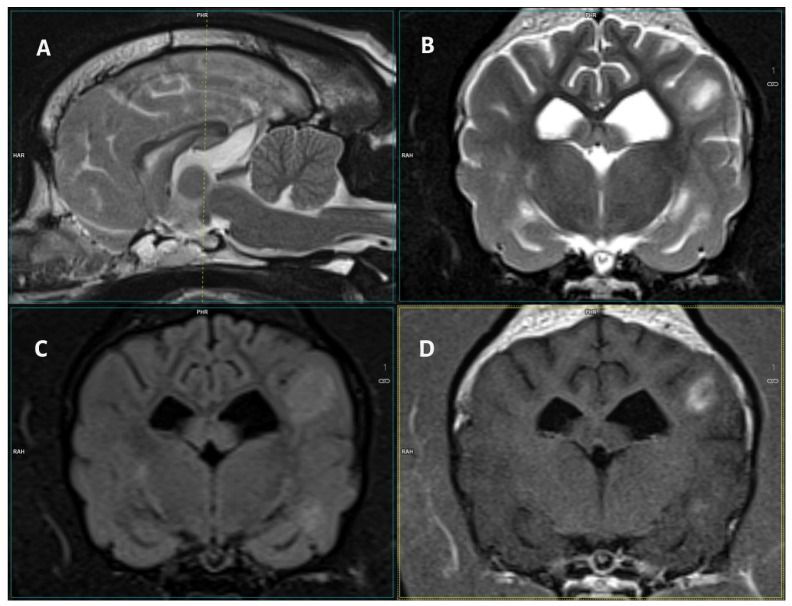
Images of a 3-T MRI study of a 5-year-old male French Bulldog. (**A**) Mid-sagittal T2-W, (**B**) T2-W transverse, (**C**) FLAIR, (**D**) T1 post-contrast transverse images of the brain. At the level of the caudal margin of the interthalamic adhesion, in the left parietal and temporal regions, there are diffuse T2-W and FLAIR hyperintense lesions with cloudy margins. They display clear, inhomogeneous contrast enhancement. In addition, partial obliteration of the sulci is observed in correspondence to the lesions.

The lesions were mostly T2-W hyperintense, FLAIR/STIR hyperintense, and T1-W isointense, showing contrast enhancement (Table 3, Figure 1 and Figure 2). A total of 44.4% (*n* = 12) of dogs displayed meningeal contrast enhancement.

In 29.6% (*n* = 8) of dogs, signs of mass effect and brain compression were described. In 44.4% (*n* = 12), there were signs of perilesional parenchymal brain edema. Midline shift was observed in 11.1% (*n* = 3) of dogs. Two dogs (7.4%) displayed cerebellar herniation through the foramen magnum.

Cerebrospinal fluid samples were taken in 85.2% (*n* = 23) of dogs, and the samples were considered normal in 34.8% of these. The median number of nucleated cells in 1 µL of CSF was 7 cells (IQR from 5 to 33). CSF cytology was performed on all dogs that received CSF examination, apart for one dog (*n* = 22, 81.5%). Of them, 13 (59.1%) exhibited a lymphocytic pleocytosis. Two dogs (9.1%) displayed monocytic and one (4.5%) exhibited neutrophilic pleocytosis. The results are summarized in Table 4.

The median time between the onset of clinical signs and diagnosis in the dogs was 5.5 (IQR 2–11.75) days. The maximal time between onset and diagnosis was 6 months (180 days) in one dog.

### 3.4. Treatment

In 77.8% dogs (*n* = 21), the treatment was started immediately. All dogs received treatment with glucocorticoids. A total of 59.3% (*n* = 16) of dogs were treated with cytosine arabinoside as an add-on therapy, and the used protocols included intravenous constant rate infusion (CRI) or subcutaneous injections of cytosine arabinoside at 25 mg/m^2^/h or 50 mg/m^2^, respectively [13,14]. Six dogs (22.2%) received antiepileptic treatments with phenobarbital, and three dogs (11.1%) received levetiracetam. In addition to the glucocorticoids, one dog (3.7%) was treated using azathioprine and one using cyclosporin.

The median duration of treatment was 210 (IQR 60–300) days, although during the study, eight patients (29.6%) were still on treatment.

### 3.5. Outcome

In three dogs (11.1%), the information on outcome and survival was lacking. Five (18.5%) of the dogs died during the treatment. The median survival time of the deceased FBs was 60 (IQR 46.8–953.8) days. Most (63.0%, *n* = 17) of the dogs were still alive at the time of the study data collection (May of 2022) or last follow-up. In these dogs, the median time from diagnosis to the last follow-up or data collection was 775 days (IQR 357.3–1331.3).The relationships between the outcome of the disease at the time of the study and the potential prognostic features are summarized in Table 3. Dogs that did not have lesions in the brainstem were more likely to be alive at the time of the study (*n* = 11/17, 64.7%) (*p* > 0.05). Dogs that did not show brain compression or mass effect on MRI were more likely to be alive at the time of the study (*n* = 12/19, 63.2%) (*p* > 0.05). Neither relationship was statistically significant (*p* > 0.05).

Additionally, some statistically significant relationships were found (flagged with * in Table 3). Out of 27 dogs, 3 had a midline shift, and all of them died during the study (*n* = 3/3, 100%) (*p* < 0.05). Dogs that presented with seizures were more likely to be deceased at the time of the study (*n* = 3/5, 60%) (*p* < 0.05). Dogs that did not experience seizures before diagnosis were more likely to be alive during data collection (*n* = 15/22, 68.2%) (*p* < 0.05). Dogs that did not relapse were more likely to be alive during the study conduction (*p* < 0.05) (see Table 5).

Out of 27 dogs, 15 (55.6%) experienced a relapse of MUO. The median time from diagnosis to relapse was 270 (IQR 105–515) days. Eleven dogs (73.3%) that relapsed were still on medication at the time of the study, and four dogs (26.7%) were no longer on medication. The median time from treatment discontinuation to relapse was 179.5 (IQR 32.8–382.5) days. In those that relapsed while on therapy (*n* = 11, 73.3%), 10 (90.9%) were being treated with prednisolone, and one (9.1%) with cyclosporine. In addition to these drugs, five dogs (45.5%) were being treated with cytarabine.

The neurological signs presented during the relapse are summarized in Table 4. The most common neurological signs during the relapse were epileptic seizures, ataxia, and depressed mentation (Table 6).

In one-third of dogs (*n* = 5/15) that relapsed, MRI scans were repeated. In two dogs, the spinal cord was affected, and in the another two dogs, forebrain lesions were observed. In one dog, the MRI study was considered negative. The lesions appeared at the same anatomical location and were inferior in severity when compared to those in the first MRI scan in 2/4 (50.0%) and were considered stable in 1/4 (25.5%) dogs. One dog (25.0%) had a severe worsening of MUO lesions on MRI. Instead of previous optic nerve involvement, inflammatory lesions were distributed throughout the thalamus, mesencephalon on the left and cortically in the parietal, occipital, and temporal lobes, causing perilesional edema and midline shift.

The dogs that experienced relapses were more likely to display multifocal lesions on the initial MRI (*n* = 11/15, 73.3%) (*p* > 0.05). Out of 10 dogs that did not relapse, only 3 died (30%), while 7 (70%) remained alive. Out of 15 dogs that did relapse, 6 died (40%), and 9 (60%) survived, demonstrating that mortality was higher within relapsed French Bulldogs (*p* < 0.05) (see Table 3).

## 4. Discussion

MUO can affect all dog breeds. However, certain breed-specific tendencies have been described [1]. In order to characterize FB presentation of MUO, first we analyzed the main complaints and the most common neurological signs with which the dogs presented. On presentation, MUO-affected French Bulldogs showed mostly forebrain signs. As previously described, signs commonly included epileptic seizures, obtunded mental status, circling, ataxia, vestibular signs, and blindness [4,15]. Cervical pain was one of the most common presentations (25.9%, *n* = 7), and the cervical spinal cord was the second most affected area (40.7%, *n* = 11) on MRI. Blindness was as common as cervical pain (25.9%, *n* = 7), and a similar number (18.5%, *n* = 5) of bulldogs displayed optic tract or retinal involvement. The most common signs in FB with MUO were directly correlated with the CNS areas affected on MRI.

In this study, we opted to involve dogs with normal CSF examination, if MRI was suggestive of MUO. More than two-thirds (34.8%, *n* = 8/23) of dogs exhibited a normal TNCC count within the CSF. This inclusion criteria allowed us to include cases in which the CSF did not completely represent the disease. Studies suggest that the cell count varies greatly in the CSF of MUO-affected dogs and can be normal in up to 57% of cases [5,10]. However, understanding the limited sensitivity of MRI to detect inflammatory lesions [6,10], we cannot exclude the possibility that some of the FB in our study were actually affected by neoplastic or vascular pathologies.

Confirmation of an exact MUO subtype can only be reached after performing histopathological examination of the affected nervous tissue. Based on the scientific literature, FBs seem to be mostly affected by necrotizing leukoencephalitis (NLE), as is also the case for the Yorkshire Terrier breed [2,4,7,8,9,12]. A study that investigated breed-specific findings of necrotizing encephalitis reported that NLE lesions in FBs on MRI seem to be less severe than those of the Yorkshire Terriers, can vary greatly in form and shape, and can involve different locations of the brain, as well as the optic nerves and retina [1]. Nearly one-fifth (18.5%) of the bulldogs in our study exhibited the involvement of the visual pathways on MRI, as described previously [1,4].

MUO displays a wide variety of presentations, which makes its recognition and early therapeutic intervention challenging. Classification into subtypes would allow for a clearer description of the MUO findings and improve accuracy, making the diagnostic process timely, efficient, and more precise. Multiple studies have previously aimed to describe different MUO subtypes on MRI. It was shown that in NLE, malacic lesions are predominately observed in the cerebral white matter and subcortical region, as well as in the brainstem and thalamus [2,4,7]. Our study results show that 52% of bulldogs showed lesions in both the white and gray matter, 26% displayed selective gray, and only 11% exhibited selective white matter lesions. Regarding meningeal involvement, mass effect, perilesional brain edema, and midline shift, NLE rarely seems to be the cause [1,4]. GME, on the other hand, is consistent with increased intracranial pressure and may present mass effect and perilesional edema [16]. NLE affects mostly white brain matter and causes minimal meningeal involvement, mass effect, or brain herniations on MRI. In our study, less than half (44.4%) of dogs displayed meningeal contrast enhancement, 11.1% of dogs showed the presence of midline shift, and two dogs (7.4%) exhibited cerebellar herniation through the foramen magnum. Only 11% of FB cases exhibited white matter lesions exclusively. Our findings suggest that the prevalence of NLE in FBs might not be as high as suspected, and that the current study possibly included FBs with different MUO subtypes and not necessarily mostly NLE. This hypothesis can be partially justified by an incredible similarity between our FB MRI findings and the overall MRI findings of MUO. A recent study by Gonçalves et al., 2024, reports that on MRI, presumed MUO lesions were mostly multifocal, 11.6% of dogs showed signs of mass effect, and 73.9% exhibited contrast enhancing parenchymal lesions, while 44.9% displayed meningeal enhancement [17]. In our study, 66.7% of the lesions were multifocal, 29.6% exhibited mass effect, 66.7% showed parenchymal contrast enhancement, and 44.4% displayed meningeal contrast uptake.

These findings could be explained by the hypothesis that FBs might be affected by a mixture of histologically confirmed MUO subtypes. A recent histopathologic case series concluded that different subtypes of MUO can contribute to the lesions and can be found together in one patient. It remains unknown whether the overlapping of different features indicates a different stage or manifestation of the same disease or a completely different pathology [7]. However, it is important to note that MRI sensitivity for detecting different MUO subtypes is low. Wolff et al. concluded that MRI is around 50% sensitive for GME detection and 46.7% sensitive for NE lesions [6].

We observed statistically significant associations between survival and different clinical features in FB. In our opinion, two relationships could be of significance in this study. Both seizures at presentation and relapsing were linked to dogs that survived for shorter periods of time. Previous studies agree with our results and confirm that dogs with MUO presenting with seizures showed a significantly reduced survival time [4,5]. Whether this is related to a more severe form of the disease or to a complication related to the presence of seizures itself is not yet clarified. In veterinary medicine, early disease detection strongly depends on the owners’ observational skills and ability to recognize early signs, as well as their overall education on animal healthcare. In our study the median time between the onset of clinical signs and diagnosis was 5.5 days, but in some dogs, as much as 180 days had passed before the owners sought an examination.

Lowrie et al. state that 65% of dogs with MUO had relapsed, and the relapse did not affect the outcome [18]. In the current study, 55.6% of dogs relapsed, and this condition was associated with a poorer outcome. A total of 60% of deceased bulldogs had relapsed at least once. Therefore, our study suggests that both epileptic seizures at presentation and relapsing might be prognostic factors in FBs with MUO, causing a shorter survival time. Additional studies are needed to confirm the potential prognostic features.

Multiple studies aim to link different disease findings with outcomes and prognosis. Oliphant et al. (2017) determined that the finding of brain parenchyma midline shift on MRI scans was not linked to poorer prognosis in dogs with MUO. Instead, older dogs and higher total nucleated cell counts on CSF were negatively correlated with survival [19]. Lowrie et al. (2013), on the other hand, concluded that the loss of the cerebral sulci and cerebellar herniation through the foramen magnum increased the risk of mortality in dogs [18]. The findings regarding the survival of dogs with indirect signs of increased intracranial pressure are controversial. In our study, dogs with the presence of the midline shift on MRI study were more likely to be deceased. All dogs with midline shift died or were euthanized during the study. Hence, the presence of midline shift on MRI could increase the risk of mortality in French Bulldogs with MUO.

Based on the current literature, overall, dogs with MUO tend to have a good chance of survival if they survive the first year. This could imply that the initial treatment phase is very important, and quick progression of the disease could indicate a negative outcome. A total of 15% of dogs with GME die before starting treatment [5,20]. Smith et al., 2009, concluded that most animals that survived the first month displayed a reasonably good chance of survival [21]. In another study, more than half of the dogs died within the first 52 days after the diagnosis, and the remaining dogs showed a good overall survival rate [18]. A similar tendency was noticed in our study, with 18.5% of dogs that died early in the course of the disease (within a median of 60 days), while the remaining two-thirds of the dogs displayed a good overall survival rate. It is speculated, therefore, that 18.5% of dogs that died early exhibited a rapid progression of the pathology, a more aggressive MUO form, or that they were already in the advanced stages of MUO.

We acknowledge that our study has several limitations. The diagnosis of MUO was presumed in all 27 dogs included in the study, as the histopathological examination was not performed. M. Lowrie, 2023, suggests the importance of randomized controlled trials to accurately evaluate treatment efficacy and survival validity in dogs affected by MUO [22]. Some of the data in our study were unavailable due to the retrospective study design, i.e., lost follow-ups or dogs still being alive at the time of the study. Three dogs received the diagnosis of the disease in one of four referral neurology centers, but were later followed by local veterinary doctors. This led to unknown survival times in some patients, making it impossible to reasonably evaluate their overall survival. Secondly, to increase the sample size and statistical relevance, we chose to perform a multi-center study. This means that a uniform diagnostic (i.e., lack of infectious disease testing for some dogs) and treatment protocol could not possibly be followed, and MUO management varied slightly. Differences in diagnostic imaging equipment and therapeutic protocols could have affected the study result reliability, making it difficult to accurately evaluate diagnostics, treatment efficacy, and survival time. It could also have led to unpredictable relapsing and differing outcomes. Finally, the number of cases collected might not necessarily represent the disease within the FB breed. Reduced statistical power and increased possibility of statistical errors were possible due to a small sample size.

## 5. Conclusions

In conclusion, the findings of the current case series suggest that FBs with MUO might have a tendency to present with acute blindness and/or cervical pain, although these are neurological signs commonly found in dogs affected by MUO. The MRI features look similar to those observed mainly in MUO-affected dogs. Seizures at presentation were correlated with multifocal MUO lesions and lower survival rates. Brainstem lesions, brain compression, mass effect, and midline shift were considered risk factors for higher mortality. Further studies are needed to confirm our findings.

## Figures and Tables

**Table 1:** Summary of clinical signs of 27 French Bulldogs with a presumptive diagnosis of MUO at presentation.

Signs at the Presentation	Percentage of Affected Dogs (%)	*n* =
Blindness	25.9	7
Cervical Pain	25.9	7
Seizures	18.5	5
Head Tilt	14.8	4
Tetraparesis	11.1	3
Pleurothotonus	7.4	2
Compulsive Behavior	7.4	2
Circling	7.4	2
Strabismus	7.4	2
Nystagmus	3.7	1

**Table 2:** Summary of neuroanatomical locations in 27 French Bulldogs with a presumptive diagnosis of MUO.

Lesion Location	Percentage of Affected Dogs (%)	*n* =
Forebrain	66.7	18
Spinal cord	40.7	11
Brainstem	37.0	10
Optic tracts	18.5	5
Cerebellum	3.7	1

**Table 3:** Summary of MRI signal characteristics in 27 French Bulldogs with presumed MUO.

Sequence	Signal Intensity	Percentage (%)	*n* =
T2-W	hyperintensity	92.6	25
	isointensity	7.4	2
FLAIR/STIR	hyperintensity	92.6	25
	isointensity	3.7	1
	hypointensity	3.7	1
T1-W	isointensity	59.3	16
	hypointensity	40.7	11
Post-contrast	enhancement	66.7	18
	meningeal enhancement	44.4	12
	non-enhancing	33.3	9

**Table 4:** Summary of CSF examination in 27 French Bulldogs with presumed MUO.

Dog Number	CSF Collection	Number of Cells	Pleocytosis Type
1	yes	35	lymphocytic
2	yes	11	lymphocytic
3	yes	26	lymphocytic
4	no		
5	yes	18	lymphocytic
6	yes	45	lymphocytic
7	yes	262	monocytic
8	yes	9	lymphocytic
9	yes	25	lymphocytic
10	yes	5	lymphocytic
11	yes	7	lymphocytic
12	yes	1	normal
13	yes	5	normal
14	yes	33	lymphocytic
15	yes	0	normal
16	yes	530	
17	yes	6	lymphocytic
18	yes	5	lymphocytic
19	yes	6	monocytic
20	yes	7	rare macrophages
21	yes	600	neutrophilic
22	no		
23	yes	0	normal
24	no		
25	no		
26	yes	2	lymphocytic
27	yes	0	normal

**Table 5:** Relationships between outcome and potential prognostic features of survival.

Prognostic Feature		Deceased	Alive
Seizures at presentation *	Present	3; 60.0%	1; 20.0%
	Absent	7; 31.8%	15; 68.2%
Midline shift *	Present	3; 100%	0; 0%
	Absent	7; 29.2%	16; 66.7%
Mass effect and compression	Present	4; 50.0%	4; 50.0%
	Absent	6; 31.6%	12; 63.2%
Brainstem lesion	Present	4; 40.0%	5; 50.0%
	Absent	6; 35.3%	11; 64.7%
Relapses *	Present	6; 40.0%	9; 60.0%
	Absent	3; 30.0%	7; 70.0%

* Data statistically significant (*p* < 0.05).

**Table 6:** Summary of clinical signs that French Bulldogs with presumed MUO presented with at the time of relapse.

Signs During Relapse	Percentage of Affected Dogs (%)	*n* =
Seizures	26.7	4
Ataxia	26.7	4
Depression	26.7	4
Blindness	13.3	2
Compulsive Behavior	13.3	2
Vestibular Syndrome	6.7	1
Tetraparesis	6.7	1
Circling	6.7	1
Cervical Pain	6.7	1

## Data Availability

The data presented in this study are available on request from the corresponding author due to (specify the reason for the restriction).

## References

[1] Flegel T. (2017). Breed-specific magnetic resonance imaging characteristics of necrotizing encephalitis in dogs. Front. Vet. Sci..

[2] Park E.S., Uchida K., Nakayama H. (2012). Comprehensive immunohistochemical studies on canine necrotizing meningoencephalitis (NME), necrotizing leukoencephalitis (NLE), and granulomatous meningoencephalomyelitis (GME). Vet. Pathol..

[3] Vandevelde M. (1980). Primary reticulosis of the central nervous system. Vet. Clin. N. Am. Small Anim. Pract..

[4] Coates J.R., Jeffery N.D. (2014). Perspectives on meningoencephalomyelitis of unknown origin. Vet. Clin. N. Am. Small Anim. Pract..

[5] Cornelis I., Van Ham L., Gielen I., De Decker S., Bhatti S.F. (2019). Clinical presentation, diagnostic findings, prognostic factors, treatment and outcome in dogs with meningoencephalomyelitis of unknown origin: A review. Vet. J..

[6] Wolff C.A., Holmes S.P., Young B.D., Chen A.V., Kent M., Platt S.R., Savage M.Y., Schatzberg S.J., Fosgate G.T., Levine J.M. (2012). Magnetic resonance imaging for the differentiation of neoplastic, inflammatory, and cerebrovascular brain disease in dogs. J. Vet. Intern. Med..

[7] Nessler J.N., Oevermann A., Schawacht M., Gerhauser I., Spitzbarth I., Bittermann S., Steffen F., Schmidt M.J., Tipold A. (2022). Concomitant necrotizing encephalitis and granulomatous meningoencephalitis in four toy breed dogs. Front. Vet. Sci..

[8] Timmann D., Konar M., Howard J., Vandevelde M. (2007). Necrotising encephalitis in a French bulldog. J. Small Anim. Pract..

[9] Young B.D., Levine J.M., Fosgate G.T., De Lahunta A., Flegel T., Matiasek K., Miller A., Silver G., Sharp N., Greer K. (2009). Magnetic resonance imaging characteristics of necrotizing meningoencephalitis in Pug dogs. J. Vet. Intern. Med..

[10] Granger N., Smith P.M., Jeffery N.D. (2010). Clinical findings and treatment of non-infectious meningoencephalomyelitis in dogs: A systematic review of 457 published cases from 1962 to 2008. Vet. J..

[11] Mayousse V., Desquilbet L., Jeandel A., Blot S. (2017). Prevalence of neurological disorders in French bulldog: A retrospective study of 343 cases (2002–2016). BMC Vet. Res..

[12] Spitzbarth I., Schenk H.C., Tipold A., Beineke A. (2010). Immunohistochemical characterization of inflammatory and glial responses in a case of necrotizing leucoencephalitis in a French bulldog. J. Comp. Pathol..

[13] Early P.J., Crook K.I., Williams L.M., Davis E.G., Muñana K.R., Papich M.G., Messenger K.M. (2017). Plasma and serum concentrations of cytarabine administered via continuous intravenous infusion to dogs with meningoencephalomyelitis of unknown etiology. J. Vet. Pharmacol. Ther..

[14] Pastina B., Early P.J., Bergman R.L., Nettifee J., Maller A., Bray K.Y., Waldron R.J., Castel A.M., Munana K.R., Papich M.G. (2018). The pharmacokinetics of cytarabine administered subcutaneously, combined with prednisone, in dogs with meningoencephalomyelitis of unknown etiology. J. Vet. Pharmacol. Ther..

[15] Talarico L.R., Schatzberg S.J. (2010). Idiopathic granulomatous and necrotising inflammatory disorders of the canine central nervous system: A review and future perspectives. J. Small Anim. Pract..

[16] Cherubini G.B., Platt S.R., Anderson T.J., Rusbridge C., Lorenzo V., Mantis P., Cappello R. (2006). Characteristics of magnetic resonance images of granulomatous meningoencephalomyelitis in 11 dogs. Vet. Rec..

[17] Gonçalves R., De Decker S., Walmsley G., Maddox T.W. (2024). Magnetic resonance imaging prognostic factors for survival and relapse in dogs with meningoencephalitis of unknown origin. Front. Vet. Sci..

[18] Lowrie M., Smith P.M., Garosi L. (2013). Meningoencephalitis of unknown origin: Investigation of prognostic factors and outcome using a standard treatment protocol. Vet. Rec..

[19] Oliphant B.J., Barnes Heller H.L., White J.M. (2017). Retrospective study evaluating associations between midline brain shift on magnetic resonance imaging and survival in dogs diagnosed with meningoencephalitis of unknown etiology. Vet. Radiol. Ultrasound.

[20] Stee K., Broeckx B.J., Targett M., Gomes S.A., Lowrie M. (2020). Cytosine arabinoside constant rate infusion without subsequent subcutaneous injections for the treatment of dogs with meningoencephalomyelitis of unknown origin. Vet. Rec..

[21] Smith P.M., Stalin C.E., Shaw D., Granger N., Jeffery N.D. (2009). Comparison of two regimens for the treatment of meningoencephalomyelitis of unknown etiology. J. Vet. Intern. Med..

[22] Lowrie M. (2023). In search of the best analysis regarding treatment for meningoencephalitis of unknown origin in dogs. Front. Vet. Sci..

[23] Burbaite E., Fiorentino E., Gallucci A., Tirrito F., Gandini G., Menchetti M. Clinical Presentation, Diagnostic Findings and Outcome in 27 French Bulldogs Diagnosed with Presumed Meningoencephalomyelitis of Unknown Origin. Proceedings of the 35th ESVN-ECVN Symposium.

